# The use of angiotensin-converting enzyme inhibitors vs. angiotensin receptor blockers and cognitive decline in Alzheimer’s disease: the importance of blood-brain barrier penetration and *APOE* ε4 carrier status

**DOI:** 10.1186/s13195-021-00778-8

**Published:** 2021-02-11

**Authors:** Michael Ouk, Che-Yuan Wu, Jennifer S. Rabin, Aaron Jackson, Jodi D. Edwards, Joel Ramirez, Mario Masellis, Richard H. Swartz, Nathan Herrmann, Krista L. Lanctôt, Sandra E. Black, Walter Swardfager

**Affiliations:** 1 Department of Pharmacology & Toxicology Room 4207, University of Toronto, Medical Sciences Building 1 King’s College Circle, Toronto, ON M5S 1A8 Canada; 2 Hurvitz Brain Sciences Program, Sunnybrook Research Institute, Toronto, ON Canada; 3 Harquail Centre for Neuromodulation, Sunnybrook Research Institute, Toronto, M4N 3M5 Canada; 4 Department of Medicine (Neurology), Sunnybrook Health Sciences Centre, University of Toronto, Toronto, ON Canada; 5 Rehabilitation Sciences Institute, University of Toronto, Toronto, ON Canada; 6 University of Ottawa Heart Institute, University of Ottawa, Ottawa, ON Canada; 7 School of Epidemiology and Public Health, University of Ottawa, Ottawa, ON Canada; 8 ICES, Ottawa, ON Canada; 9 Canadian Partnership for Stroke Recovery, Toronto, ON Canada; 10 Department of Psychiatry, Sunnybrook Health Sciences Centre, University of Toronto, Toronto, ON Canada; 11 KITE UHN Toronto Rehabilitation Institute, Toronto, ON Canada

**Keywords:** Alzheimer’s disease, Hypertension, Angiotensin receptor blockers, Angiotensin-converting enzyme inhibitors, Cognition, Memory

## Abstract

**Background:**

The antihypertensive angiotensin receptor blockers (ARBs) and angiotensin-converting enzyme inhibitors (ACE-Is) have similar indications and mechanisms of action, but prior work suggests divergence in their effects on cognition.

**Methods:**

Participants in the National Alzheimer’s Coordinating Center database with a clinical diagnosis of dementia due to Alzheimer’s disease (AD) using an ACE-I or an ARB at any visit were selected. The primary outcome was delayed recall memory on the Wechsler Memory Scale Revised – Logical Memory IIA. Other cognitive domains were explored, including attention and psychomotor processing speed (Trail Making Test [TMT]-A and Digit Symbol Substitution Test [DSST]), executive function (TMT-B), and language and semantic verbal fluency (Animal Naming, Vegetable Naming, and Boston Naming Tests). Random slopes mixed-effects models with inverse probability of treatment weighting were used, yielding rate ratios (RR) or regression coefficients (B), as appropriate to the distribution of the data. Apolipoprotein (*APOE)* ε4 status and blood-brain barrier (BBB) penetrance were investigated as effect modifiers.

**Results:**

Among 1689 participants with AD, ARB use (*n* = 578) was associated with 9.4% slower decline in delayed recall performance over a mean follow-up of 2.28 years compared with ACE-I use (*n* = 1111) [RR = 1.094, *p* = 0.0327]; specifically, users of BBB-crossing ARBs (RR = 1.25, *p* = 0.002), BBB-crossing ACE-Is (RR = 1.16, *p* = 0.010), and non-BBB-crossing ARBs (RR = 1.20, *p* = 0.005) had better delayed recall performance over time compared with non-BBB-crossing ACE-I users. An interaction with *APOE* ε4 status (drug × *APOE* × time RR = 1.196, *p* = 0.033) emerged; ARBs were associated with better delayed recall scores over time than ACE-Is in non-carriers (RR = 1.200, *p* = 0.003), but not in carriers (RR = 1.003, *p* = 0.957). ARB use was also associated with better performance over time on the TMT-A (*B* = 2.023 s, *p* = 0.0004) and the DSST (*B* = 0.573 symbols, *p* = 0.0485), and these differences were significant among *APOE* ε4 non-carriers (*B* = 4.066 s, *p* = 0.0004; and *B* = 0.982 symbols, *p* = 0.0230; respectively). Some differences were seen also in language and verbal fluency among *APOE* ε4 non-carriers.

**Conclusions:**

Among *APOE* ε4 non-carriers with AD, ARB use was associated with greater preservation of memory and attention/psychomotor processing speed, particularly compared to ACE-Is that do not cross the blood-brain-barrier.

**Supplementary Information:**

The online version contains supplementary material available at 10.1186/s13195-021-00778-8.

## Background

Hypertension currently affects roughly two thirds of all Americans aged 65 or older [1], and its burden has steadily increased in past decades [2]. In addition to being a major contributor to cardiovascular disease risk and mortality, hypertension has recently been established as a significant independent risk factor for cognitive decline and Alzheimer’s disease (AD) dementia [3–5]. Individuals with hypertension have been shown to exhibit poorer performance in multiple cognitive domains, including memory, psychomotor processing speed, attention, and executive function [6–8]. Consequently, the relationships between the use of antihypertensive medications with dementia incidence and cognitive decline have become an important area of research. Some evidence has shown that reductions in cerebral blood flow associated with hypertension can be reversed through antihypertensive treatment, potentially mitigating cognitive and functional decline associated with AD [9, 10]. However, the results have been variable; while the SPRINT-MIND randomized clinical trial recently associated intensive blood pressure (BP) control with a reduced risk of mild cognitive impairment or probable dementia [11], and several observational studies have associated the use of any antihypertensive agent with a reduced risk of incident dementia or cognitive decline [12–15], others have identified no significant benefits on one or both outcomes [16–18].

Angiotensin II receptor blockers (ARBs) and angiotensin-converting enzyme inhibitors (ACE-Is) are first-line treatment options for hypertension which have similar indications and safety profiles [19]. Mechanistically, they both act upon targets within the renin-angiotensin-aldosterone system (RAAS) to elicit their blood pressure-lowering effects, with ARBs acting at the angiotensin II type-1 receptors (AT1Rs), and ACE-Is acting upstream at the angiotensin-converting enzyme-1 (ACE-1) [20]. Despite these similarities, a growing body of literature suggests that these antihypertensive classes may differ in their neuroprotective effects. Observational studies have found both ARBs and ACE-Is to be independently associated with reduced cognitive decline and incident dementia [21–24]; in contrast, the literature in toto has been met with mixed conclusions [12, 16], which may be due in part to heterogeneity in the study populations. Direct head-to-head comparisons of ARBs vs. ACE-Is have been limited, but studies have associated ARB use with less brain atrophy [25, 26], lower dementia incidence [23, 27–29], and slower cognitive decline [25, 30, 31] relative to ACE-I use. In a previous pathology study, ARB use was associated with fewer plaques and tangles than ACE-I use, suggesting that these agents may act differently on AD pathological development [32] and that therefore their effects might be examined specifically in the context of AD [33].

This longitudinal study aimed to compare users of ACE-Is vs. ARBs with a diagnosis of AD dementia on memory and other cognitive outcomes over time. Taking advantage of a relatively large sample size from the National Alzheimer’s Coordinating Center (NACC) database [34], the study further aimed to elucidate factors that may have contributed to heterogeneity in the existing body of literature. Specifically, apolipoprotein (*APOE*) ε4 allele carrier status was examined due to its established role as strongest genetic risk factor for AD, in addition to previous evidence supporting associations between *APOE* genotype and neurological outcomes among users of ARBs or ACE-Is [35]. Furthermore, given conflicting evidence that the ability of these drugs to penetrate the blood-brain barrier (BBB) may be integral to their neurological benefits [24, 35, 36], we further compared BBB-crossing and non-BBB-crossing ARBs and ACE-Is.

## Methods

### Data source

The National Alzheimer’s Coordinating Center (NACC) was established in 1999 by the National Institute on Aging/NIH (U01 AG016976) to facilitate collaborative research. The NACC database consists of longitudinal participant data from approximately 39 different U.S. Alzheimer’s Disease Centers (ADCs). This analysis reflects data from the National Alzheimer’s Clinical Coordinating Center (NACC) Uniform Data Set (UDS) collected between September 2005 and June 2019. The NACC UDS collects data in a structured and standardized format across all ADCs using a prospective, longitudinal clinical evaluation. Subjects enrolled at each ADC may come from clinician referral, self-referral by patients or family members, active recruitment, or volunteering, and are best regarded as a referral-based or volunteer case series.

### Participant selection

Participants with a diagnosis of AD based on NINCDS-ADRDA criteria or NIA-AA criteria [37, 38], who met criteria for dementia and were using an ACE-I or an ARB with at least one outcome for the Wechsler Memory Scale Revised-Logical Memory Test IIA (WMS-R LM IIA)—Delayed Recall, were selected for inclusion in the analysis. Details of the participant selection process can be seen in Figure S1. Participants using both an ACE-I and an ARB simultaneously during the study period were excluded from the analysis. Furthermore, participants with a diagnosis of frontotemporal dementia, vascular dementia, Parkinson’s disease, primary progressive aphasia, a history of traumatic brain injury, cancer, or epilepsy were also excluded from the analysis.

### Drug exposures

Medication use within 2 weeks of each participant visit was identified from a structured medication inventory. Participants, or co-participants where appropriate, were asked to bring to or report all prescription medications being used currently or within 2 weeks prior to each study visit. The medication inventories were then completed by trained ADC staff or physicians. ACE-Is and ARBs were the drug classes of interest. Other antihypertensive drug classes, including beta-adrenergic antagonists (BBs), calcium channel blockers (CCBs), and diuretics (DRTCs), were also identified for inclusion as covariates in the analyses.

In addition to comparing ARBs and ACE-Is overall, we performed a secondary analysis examining the role of blood-brain barrier penetrance in moderating drug effects on cognition. For this purpose, individuals were allocated to four groups according to their prescription: (1) *users of non-BBB-crossing ARBs* [eprosartan, irbesartan, losartan, olmesartan], (2) *users of BBB-crossing ARBs* [azilsartan, candesartan, telmisartan, valsartan], (3) *users of non-BBB-crossing ACE-Is* [benazepril, enalapril, moexepril, quinapril, ramipril], and (4) *users of BBB-crossing ACE-Is* [captopril, fosinopril, lisinopril, perindopril, trandolapril, zofenopril]. We utilized available data from studies which have classified and categorized these drugs previously, predominantly on the basis of evidence from basic animal science data [39–47] and existing observational analyses [30, 35, 48–50].

### Primary outcome

The primary outcome of interest was delayed recall score assessed using the Wechsler Memory Scale Revised-Logical Memory Test IIA (WMS-R LM IIA) (scores range between 0 and 25; higher scores indicate better performance) [51]. Recall trials occurred after a 20-min delay. Delayed recall was selected as the primary outcome because it is a sensitive measure of memory and highly reflective of a cognitive domain impacted profoundly in those with AD [52, 53].

### Exploratory outcomes

Given the broader associations between hypertension and overall cognitive decline [6–8], we examined how ARBs vs. ACE-Is might impact other domains of cognition by performing comparisons of the [1] Trail Making Test (TMT) A and B (time to completion) [54], [2] WAIS-R Digit Symbol Substitution Test (DSST [number of correct symbols; scores range between 0 and 93]) [55], [3] CERAD Animal Category Fluency (total score; scores range between 0 and 77) [56], [4] Vegetable Category Fluency total score (total score; scores range between 0 and 77) [56], and [5] Boston Naming Test (total score; scores range between 0 and 30) [57] in the same subset of participants who were analyzed for delayed recall outcomes. As these were exploratory analyses, we did not adjust for multiple comparisons.

### Statistical analysis

Analyses were conducted using R (version 3.6.2), and figures were created using the ggplot2 package [58]. Descriptive statistics were generated to characterize the study cohort according to all study variables. One-way analysis of variance (ANOVA) was used to compare the groups for continuous variables, and chi-square or Fisher exact testing was used to compare the groups for nominal or categorical variables at baseline.

To quantify the associations between ARB vs. ACE-I use and longitudinal changes in delayed recall, zero-inflated negative binomial mixed-effects regression models with random slopes and intercepts were used (glmmTMB package) [59]. For the TMT-A and TMT-B, zero-inflated Gaussian variants of this model were used to accommodate the distribution of the data, with 150 s minus time-to-completion used as the outcome for the TMT-A, and 300 s minus time-to-completion used as the outcome for the TMT-B. Zero-inflated models were used to handle potential floor or ceiling effects resulting from an excess of zeroes in the outcome scores. For the WAIS-R DSST, Animal Fluency, Vegetable Fluency, and Boston Naming Test, linear mixed-effects models with random slopes and intercepts were used. For negative binomial mixed-effects models, effect sizes were reported as rate ratios (RRs), which indicate the fold-change in delayed recall score over time relative to the reference group. For zero-inflated Gaussian mixed-effects models, unstandardized regression coefficients (*B*) were reported; finally, for regular linear mixed-effects models, standardized coefficients (*β*) were used to express the magnitude of associations. For the main analyses, ACE-Is were selected as the reference group. Correction for multiple comparisons were not applied to the various permutations of BBB-crossing and non-BBB-crossing drug comparisons, as the estimates were derived from a single model with a variable reference group. All models were adjusted for clinically important covariates, including sex, baseline MMSE score, and at each study visit, age, years of education, atrial fibrillation, beta blocker use, calcium channel blocker use, diuretic use, concomitant AD medication use, systolic BP, smoking, and depression within the preceding 2 years. Additionally, models were adjusted for potential confounding by indication, through the implementation of inverse probability of treatment weighting (IPTW) based on factors selected a priori which may have influenced the likelihood to be prescribed an ARB or an ACE-I (ipw package) [60]. Specifically, marginal structural models which consider previous drug exposure were used to generate stabilized time-varying treatment probability weights based on the following factors: race, body mass index (BMI), stroke, hypercholesterolemia, diabetes, myocardial infarction, and heart failure. These factors were selected based on American hypertension management guidelines [61–63] and were ascertained using variables which existed within the UDS.

The *APOE* ε4 allele is a genetic risk factor for late-onset AD and accelerates disease progression [64, 65]. Therefore, as a further exploratory analysis, we investigated *APOE* ε4 allele carrier status (those with an ε2/ε4, ε3/ε4, or ε4/ε4 genotype) as a potential modifier of the associations between ARB or ACE-I use and cognition over time, using a drug × *APOE* ε4 × time interaction term. We then determined the conditional associations between drug class and cognitive outcomes over time in *APOE* ε4 carriers and *APOE* ε4 non-carriers.

Sensitivity analyses were considered to ensure the robustness of estimates. A post hoc model in which users of ARBs or ACE-Is who switched between the two drug classes during the study period were excluded was conducted in order to ascertain potential cross-over effects, although the marginal structural models used for propensity weighting account for previous exposures. Furthermore, because prescription practices may differ geographically, from site to site, a post hoc model was conducted with NACC ADC identifiers incorporated into the IPTW.

## Results

### Subject characteristics

Of 40,481 participants (140,861 visits conducted between September 2005 and June 2019), we identified a total of 1689 participants (3028 visits) who met criteria for inclusion with a diagnosis of AD dementia, available delayed recall outcomes, and use of an ARB or an ACE-I (participant selection process shown in Figure S1). The mean duration of follow-up did not differ significantly between users of ARBs (2.28 ± 1.48 years among the 46.5% with ≥ 2 observations, *n* = 578 at baseline, *n* = 257 BBB-crossing) and users of ACE-Is (2.27 ± 1.51 years among the 45.6% with ≥ 2 observations, *n* = 1111 at baseline, *n* = 757 BBB-crossing). Baseline characteristics are shown in Table 1. Users of ARBs and ACE-Is did not differ significantly in the prevalence of vascular risk factors, but there was a greater proportion of women and a higher MMSE score at baseline in the ARB-treated group. Additionally, a higher proportion of ARB users suffered from active depression and reported concurrent use of a CCB at baseline. These characteristics were included as covariates in all models.

**Table 1:** Baseline demographics and characteristics by diagnosis and medication class

	ARB (***n*** = 578)	ACE-I (***n*** = 1111)	***p***
**Baseline demographics**			
Follow-up time (years)	2.28 (1.48)	2.27 (1.51)	0.904
Age (years)	77.2 (8.1)	76.8 (8.6)	0.377
Female (%)	360 (62.3%)	533 (48.0%)	**< 0.001**
Race (Caucasian)	468 (81.0%)	925 (83.3%)	0.240
BMI (kg/m^2^)	27.5 (5.0)	27.1 (5.0)	0.128
Education (years)	14.3 (3.5)	14.2 (3.6)	0.577
Smoking history (years)	11.4 (16.6)	10.6 (16.4)	0.342
Systolic BP (mmHg)	139.0 (20.2)	137.8 (20.4)	0.269
Diastolic BP (mmHg)	75.6 (11.2)	74.3 (11.0)	**0.019**
**AD-related measures**			
MMSE	22.5 (4.9)	21.8 (5.4)	**0.004**
*APOE* ε4 carrier	311 (53.8%)	648 (58.3%)	0.075
AD medication use	396 (68.5%)	738 (66.4%)	0.387
**Comorbidities**			
Hypercholesterolemia	376 (65.1%)	727 (65.6%)	0.818
Hypertension	549 (95.0%)	1025 (92.3%)	0.112
Stroke/TIA history	87 (15.1%)	148 (13.3%)	0.330
Heart failure	29 (5.0%)	43 (3.9%)	0.268
Myocardial infarct	43 (7.5%)	120 (10.8%)	0.026
Diabetes	131 (22.7%)	264 (23.8%)	0.613
Depression	246 (42.6%)	408 (36.7%)	**0.019**
**Other medication use**			
β-Blockers	140 (24.2%)	308 (27.7%)	0.122
CCBs	166 (28.7%)	267 (24.0%)	**0.036**
DRTCs	220 (38.1%)	370 (33.3%)	0.052
Statins	338 (58.5%)	694 (62.5%)	0.111
Antidepressants	216 (37.4%)	407 (36.6%)	0.766
NSAIDs	262 (45.3%)	508 (45.7%)	0.877

Continuous variables and categorical variables were reported in observed/unweighted mean (SD) and proportion, respectively

### Relationships between ARB vs. ACE-I use and memory decline

**Fig. 1:**
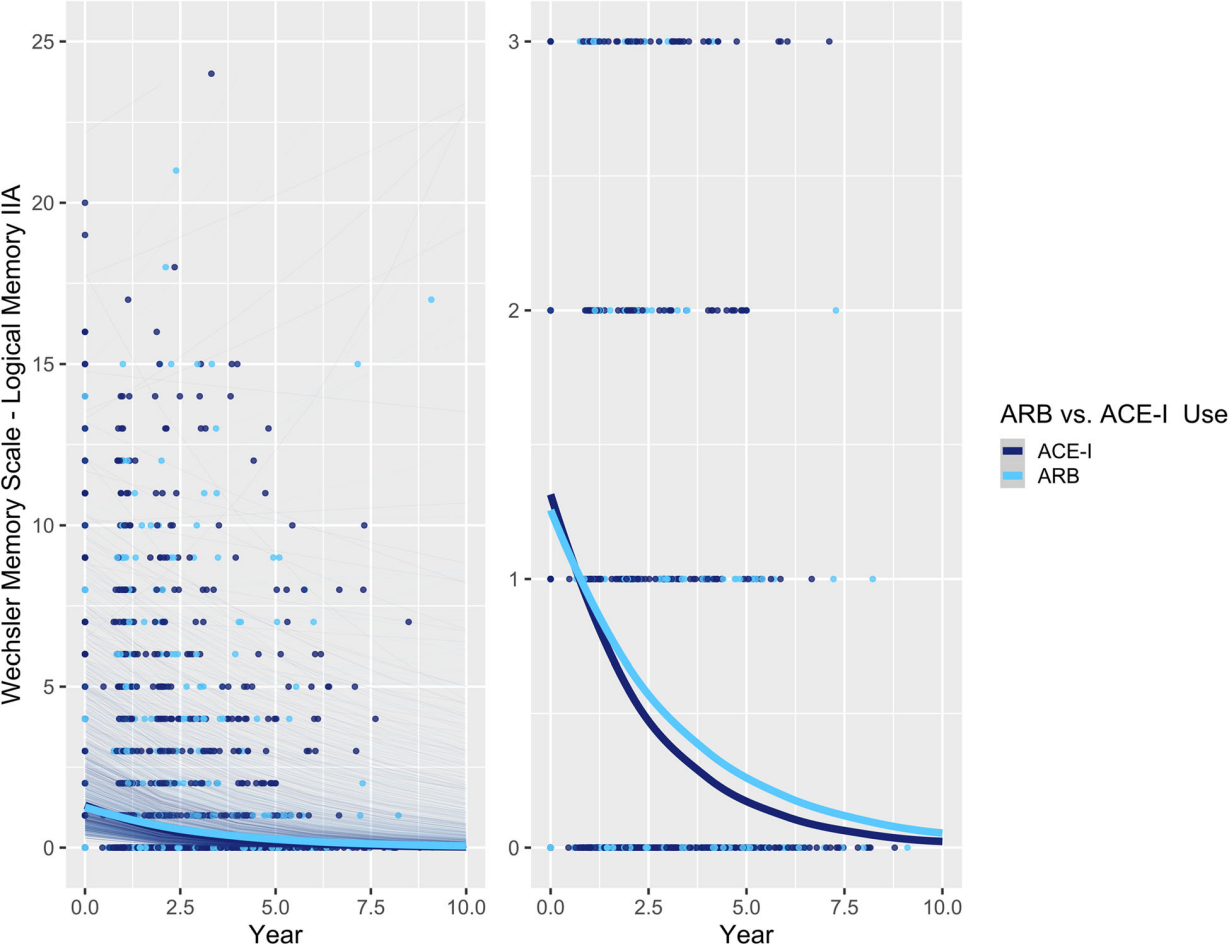
Associations between ARB vs. ACE-I use and delayed recall performance over time in participants with AD. Left: plot showing full range of outcome scores; Right: plot with reduced *y*-axis cut-off, to better show differences between ARB and ACE-I groups. Thick lines represent the total estimated association adjusted for covariates; thin lines represent estimated associations adjusted for covariates for each participant

The use of an ARB was associated with a 9.4% slower decline in delayed recall compared to the use of an ACE-I (RR [95% confidence interval] = 1.094 [1.007, 1.188], *p* = 0.0327; Table 2; Fig. 1). In a post hoc analysis excluding individuals who switched between ARBs and ACE-Is during the study period (*n* = 32), the estimate remained significant (RR = 1.099 [1.008, 1.199], *p* = 0.0323). Moreover, including NACC ADC identifiers in the IPTW model did not significantly impact the estimate (RR = 1.096 [1.008, 1.191], *p* = 0.0304).

**Table 2:** Rate ratios for relationships between WMS-R LM IIA—Delayed Recall score and ACE-I vs. ARB use over time (*n* = 1689)

	Overall			APOE ε4 non-carriers			APOE ε4 carriers		
	RR [95% CI]	***z***	***p*** value	RR [95% CI]	***z***	***p*** value	RR [95% CI]	***z***	***p*** value
**ARBs vs. ACE-Is**	1.094 [1.007, 1.188]	2.14	**0.0327**	1.200 [1.064, 1.354]	2.97	**0.0030**	1.003 [0.897, 1.122]	0.06	0.9568
**C-ARBs vs. NC-ACE-Is**	1.250 [1.089, 1.434]	3.16	**0.0015**	1.354 [1.116, 1.642]	3.08	**0.0020**	1.130 [0.932, 1.371]	1.25	0.2124
**C-ACE-Is vs. NC-ACE-Is**	1.158 [1.036, 1.293]	2.57	**0.0098**	1.168 [0.995, 1.385]	1.90	0.0571	1.140 [0.976, 1.331]	1.65	0.0993
**NC-ARBs vs. NC-ACE-Is**	1.199 [1.055, 1.365]	2.79	**0.0054**	1.324 [1.108, 1.583]	3.07	**0.0021**	1.104 [0.923, 1.321]	1.08	0.2785
**C-ARBs vs. NC-ARBs**	1.042 [0.921, 1.179]	0.65	0.5175	1.022 [0.865, 1.208]	0.26	0.7947	1.024 [0.869, 1.205]	0.28	0.7802
**C-ARBs vs. C-ACE-Is**	1.080 [0.965, 1.209]	1.34	0.1798	1.159 [0.985, 1.364]	1.78	0.0746	0.992 [0.854, 1.152]	− 0.11	0.9161

Three-way *APOE* × ARB vs. ACE-I × Time interaction: RR = 1.196 [1.015, 1.410], *z* = 2.135, ***p*** =** 0.0328**

C-ARB: BBB-crossing ARB; C-ACE-I: BBB-crossing ACE-I; NC-ARB: Non-BBB-crossing ARB; NC-ACE-I: Non-BBB-crossing ACE-I

### Relationships between ARB vs. ACE-I use and decline in performance in exploratory cognitive domains

Use of an ARB was associated with better performance on the TMT-A (*B* [95% confidence interval] = 2.023 [0.492, 3.553] seconds, *p* = 0.0096) (Table 3) and the WAIS-R DSST (*β* = 0.050 [0.001, 0.099], *p* = 0.0483, translating to 0.573 symbols) (Table 4).

**Table 3:** Relationships between Trail Making Test A performance and ACE-I vs. ARB use over time (*n* = 1601)

	Overall			APOE ε4 non-carriers			APOE ε4 carriers		
	***B*** [95% CI]	***z***	***p*** value	***B*** [95% CI]	***z***	***p*** value	***B*** [95% CI]	***z***	***p*** value
**ARBs vs. ACE-Is**	2.023 [0.492, 3.553]	2.59	**0.0096**	4.066 [1.816, 6.317]	3.54	**0.0004**	1.458 [− 0.365, 3.282]	1.57	0.1171
**C-ARBs vs. NC-ACE-Is**	3.349 [0.766. 5.933]	2.54	**0.0110**	6.086 [2.431, 9.741]	3.26	**0.0011**	2.054 [− 1.123, 5.230]	1.35	0.1785
**C-ACE-Is vs. NC-ACE-Is**	0.985 [− 0.121, 3.184]	0.88	0.3798	1.604 [− 1.061, 4.810]	0.98	0.3267	0.771 [− 1.892, 3.434]	0.57	0.5703
**NC-ARBs vs. NC-ACE-Is**	2.316 [− 0.176, 4.810]	1.82	0.0685	4.480 [0.844, 8.116]	2.42	**0.0157**	2.049 [− 0.936, 5.034]	1.27	0.2051
**C-ARBs vs. NC-ARBs**	1.033 [− 1.228, 3.293]	0.90	0.3705	1.606 [− 1.510, 4.722]	1.01	0.3125	0.005 [− 2.598, 2.608]	0.01	0.9971
**C-ARBs vs. C-ACE-Is**	2.364 [0.308, 4.420]	2.25	**0.0242**	4.482 [1.620, 7.343]	3.07	**0.0022**	1.283 [− 1.172, 3.737]	1.02	0.3058

Three-way *APOE* × ARB vs. ACE-I × Time interaction: *B* = 2.608 [− 0.300, 5.516] s, *z* = 1.76, *p* = 0.0788

C-ARB: BBB-crossing ARB; C-ACE-I: BBB-crossing ACE-I; NC-ARB: Non-BBB-crossing ARB; NC-ACE-I: Non-BBB-crossing ACE-I

**Table 4:** Relationships between WAIS-R Digit Symbol Substitution Test performance and ACE-I vs. ARB use over time (*n* = 1544)

	Overall				APOE ε4 non-carriers				APOE ε4 carriers			
	***β*** [95% CI]	*t*	df	***p*** value	***β*** [95% CI]	*t*	df	***p*** value	***β*** [95% CI]	*t*	df	***p*** value
**ARBs vs. ACE-Is**	0.050[0.001, 0.099]	1.98	533.5	**0.0483**	0.085[0.012, 0.158]	2.28	533.9	**0.0230**	0.0471[− 0.012, 0.106]	1.56	558.9	0.1186
**C-ARBs vs. NC-ACE-Is**	0.044[− 0.012, 0.101]	1.54	586.1	0.1252	0.106[0.026, 0.185]	2.60	647.9	**0.0095**	0.020 [− 0.051, 0.091]	0.55	574.0	0.5852
**C-ACE-Is vs. NC-ACE-Is**	0.012[− 0.061, 0.086]	0.33	562.0	0.7388	0.055[− 0.050, 0.161]	1.03	708.9	0.3035	− 0.023[− 0.115, 0.069]	− 0.49	487.1	0.6224
**NC-ARBs vs. NC-ACE-Is**	0.043[− 0.016, 0.102]	1.42	600.3	0.1560	0.074[− 0.009, 0.158]	1.74	694.8	0.0816	0.024[− 0.049, 0.098]	0.65	523.3	0.5177
**C-ARBs vs. NC-ARBs**	0.005[− 0.046, 0.055]	0.18	609.9	0.8567	0.037[− 0.030, 0.104]	1.08	994.6	0.2822	− 0.003[− 0.062, 0.056]	− 0.09	851.0	0.9292
**C-ARBs vs. C-ACE-Is**	0.036[− 0.010, 0.082]	1.53	853.1	0.1250	0.070[0.003, 0.136]	2.06	606.4	**0.0400**	0.035[− 0.020, 0.090]	1.24	678.5	0.2147

Three-way *APOE* × ARB vs. ACE-I × Time interaction: *β* = 0.025 [− 0.037, 0.088], *t* = 0.79, *p* = 0.4309

C-ARB: BBB-crossing ARB; C-ACE-I: BBB-crossing ACE-I; NC-ARB: Non-BBB-crossing ARB; NC-ACE-I: Non-BBB-crossing ACE-I

There were no differences between ARBs and ACE-Is in executive function (TMT-B; Table S1), nor in language (Animal Naming, Vegetable Naming, and Boston Naming tests; Tables S2, S3, S4).

### Interactions between ARB vs. ACE-Is and APOE ε4 carrier status on memory

We further examined relationships between ARB and ACE-I use separately within subgroups of *APOE* ε4 carriers and non-carriers. With respect to delayed recall, a significant 3-way interaction emerged between ARB vs. ACE-I x Time and *APOE* ε4 genotype (RR = 1.196 [1.015, 1.410], *p* = 0.0328), such that a greater benefit of ARBs relative to ACE-Is was observed among *APOE* ε4 non-carriers (RR = 1.200 [1.064, 1.354], *p* = 0.0030) (Figure S2) than among *APOE* ε4 carriers (RR = 1.003 [0.897, 1.122], *p* = 0.9568) (Figure S2).

### Interactions between ARB vs. ACE-Is and APOE ε4 carrier status on exploratory cognitive outcomes

The use of an ARB vs. an ACE-I was associated with better performance over time on the TMT-A (*B* = 4.066 [1.816, 6.317] seconds, *p* = 0.0004; Table 3) and the WAIS-R DSST (*β* = 0.085 [0.012, 0.158], *p* = 0.0230, translating to 0.982 symbols; Table 4) specifically among *APOE* ε4 non-carriers. The use of an ARB vs. an ACE-I was not associated with greater performance over time on the Animal Naming, Vegetable Naming, or Boston Naming tests, although significant differences were seen for some subgroup comparisons specifically within the *APOE* ε4 non-carriers (Tables S2, S3, S4).

### BBB-crossing vs. non-BBB-crossing ARBs and ACE-Is and memory

**Fig. 2:**
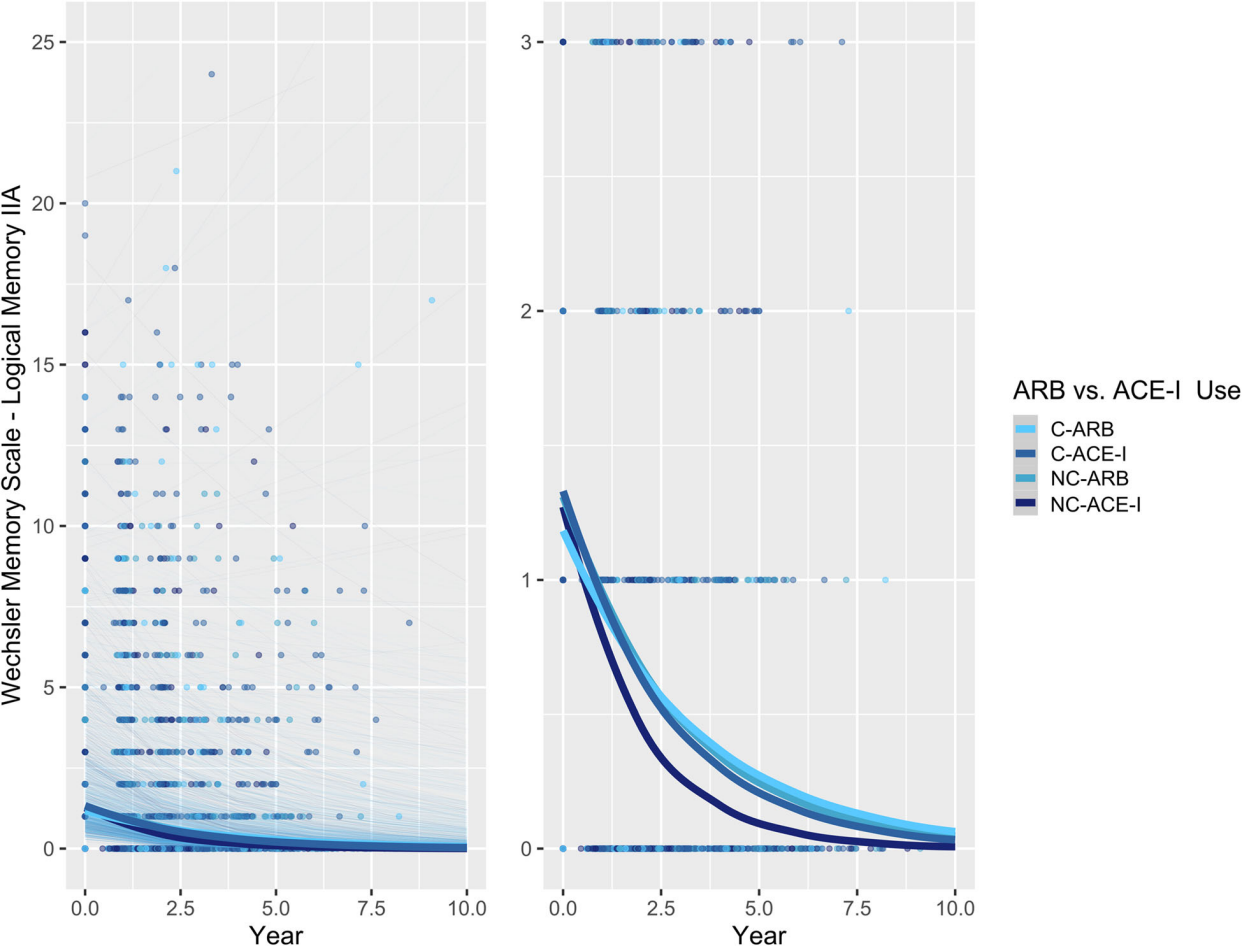
Associations between BBB-crossing and non-BBB-crossing ARBs vs. ACE-Is and delayed recall performance over time in participants with AD. Left: plot showing full range of outcome scores. Right: plot with reduced *y*-axis cut-off, to better show differences between ARB and ACE-I groups. Thick lines represent the total estimated association adjusted for covariates; thin lines represent estimated associations adjusted for covariates for each participant. C-ARB: BBB-crossing ARB; C-ACE-I: BBB-crossing ACE-I; NC-ARB: Non-BBB-crossing ARB; NC-ACE-I: Non-BBB-crossing ACE-I

A multilevel analysis was implemented to compare BBB-crossing ARBs and ACE-Is. With respect to delayed recall, among all participants with AD, BBB-crossing ARBs (RR = 1.250 [1.089, 1.434], *p* = 0.0015), non-BBB-crossing ARBs (RR = 1.199 [1.055, 1.365], *p* = 0.0054), and BBB-crossing ACE-Is (RR = 1.158 [1.036, 1.293], *p* = 0.0098) were all associated with significantly better performance over time relative to non-BBB-crossing ACE-Is (Table 2; Fig. 2). However, BBB-crossing ARBs did not differ significantly from BBB-crossing ACE-Is (RR = 1.080 [0.965, 1.209], *p* = 0.1798).

### BBB-crossing vs. non-BBB-crossing ARBs and ACE-Is and memory in APOE ε4 carriers vs. non-carriers

**Fig. 3:**
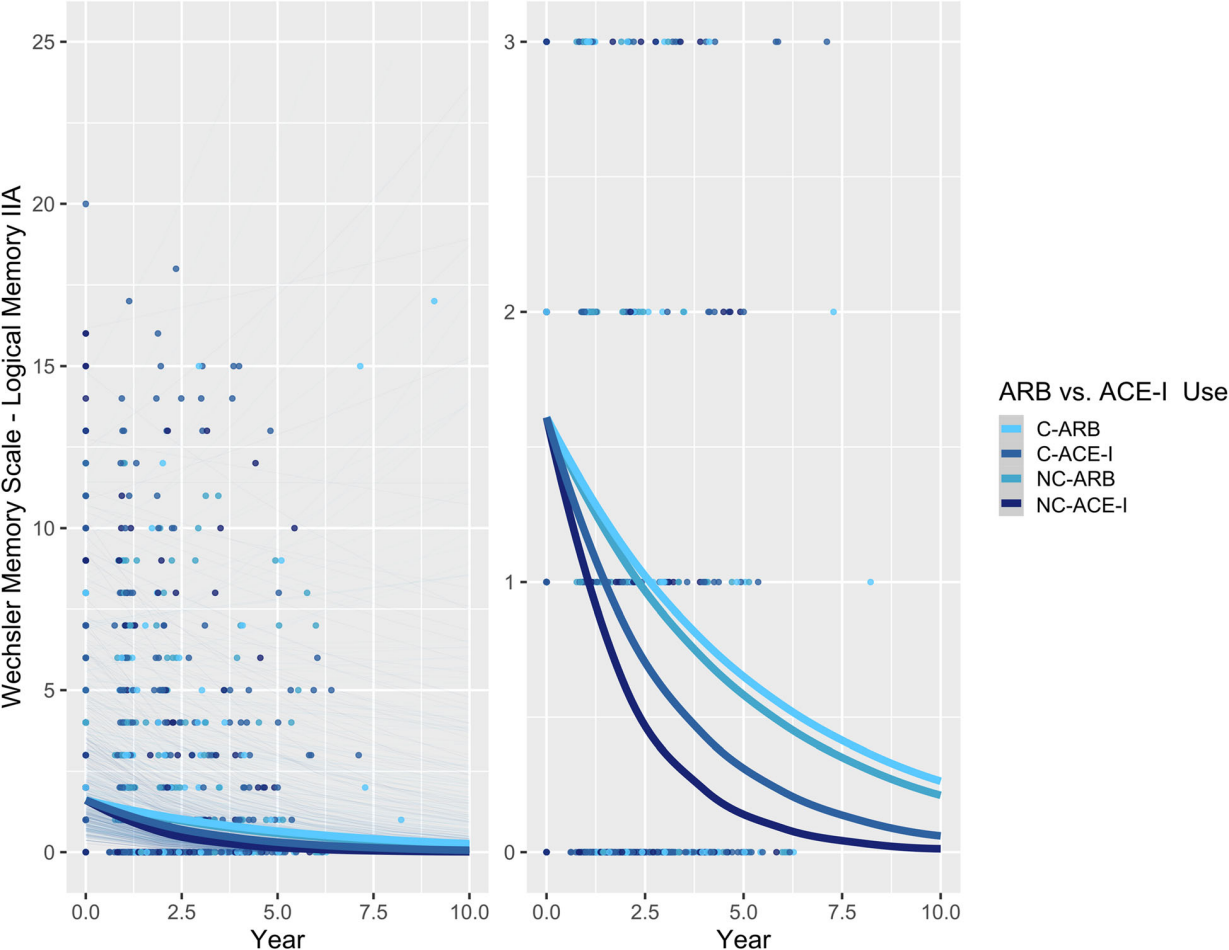
Associations between ARB vs. ACE-I use and delayed recall performance over time in non-APOE ε4 carriers with AD. Left: plot showing full range of outcome scores. Right: plot with reduced *y*-axis cut-off, to better show differences between ARB and ACE-I groups. Thick lines represent the total estimated association adjusted for covariates; thin lines represent estimated associations adjusted for covariates for each participant. C-ARB: BBB-crossing ARB; C-ACE-I: BBB-crossing ACE-I; NC-ARB: Non-BBB-crossing ARB; NC-ACE-I: Non-BBB-crossing ACE-I

In *APOE* ε4 non-carriers, both BBB-crossing ARBs (RR = 1.354 [1.116, 1.642], *p* = 0.0020) and non-BBB-crossing ARBs (RR = 1.324 [1.108, 1.583], *p* = 0.0021) were associated with significantly better memory performance relative to non-BBB-crossing ACE-Is (Table 2; Fig. 3). The BBB-crossing ACE-Is did not differ significantly from the other ARBs or ACE-Is (*p* > 0.05; Fig. 3).

**Fig. 4:**
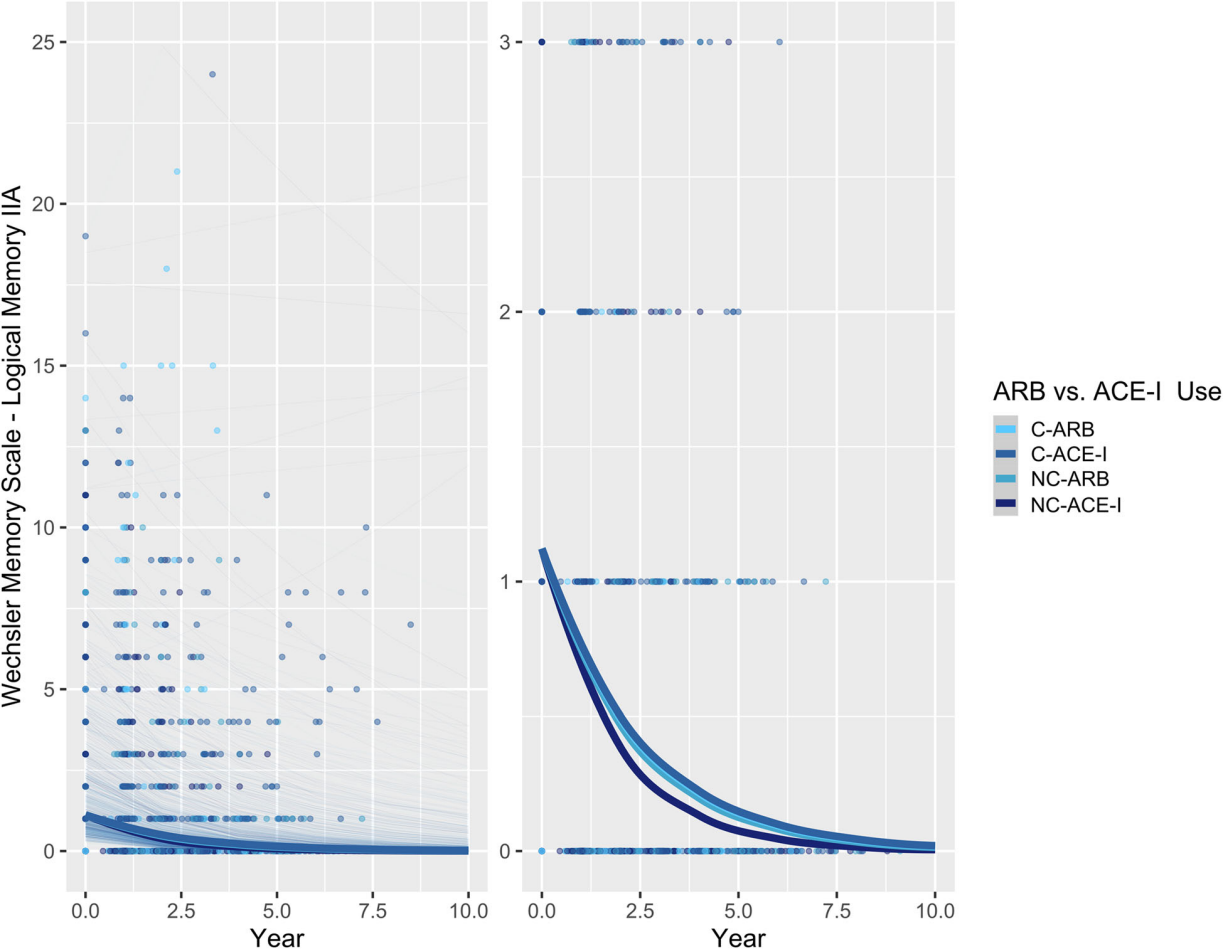
Associations between ARB vs. ACE-I use and delayed recall performance over time in APOE ε4 carriers with AD. Left: plot showing full range of outcome scores. Right: plot with reduced *y*-axis cut-off, to better show differences between ARB and ACE-I groups. Thick lines represent the total estimated association adjusted for covariates; thin lines represent estimated associations adjusted for covariates for each participant. C-ARB: BBB-crossing ARB; C-ACE-I: BBB-crossing ACE-I; NC-ARB: Non-BBB-crossing ARB; NC-ACE-I: Non-BBB-crossing ACE-I

In contrast to the *APOE* ε4 non-carriers, there were no significant differences in memory between BBB-crossing and non-crossing ARBs and ACE-Is in *APOE* ε4 carriers, although non-BBB-crossing ACE-Is were still associated with the poorest performance (Table 2; Fig. 4).

### BBB-crossing vs. non-BBB-crossing ARBs and ACE-Is and exploratory cognitive outcomes in APOE ε4 carriers vs. non-carriers

Similar to memory, *APOE* ε4 carrier status impacted the effects of ARBs vs. ACE-Is on attention, processing speed, and semantic fluency over time. No significant differences were observed in any ARB vs. ACE-I comparison among *APOE* ε4 carriers. In contrast, among *APOE* ε4 non-carriers, multiple comparisons were significant. BBB-crossing ARBs were associated with the greatest performance over time relative to non-BBB-crossing ACE-Is on the TMT-A (*B* = 6.086 [2.431, 9.741] seconds, *p* = 0.0011), DSST (*β* = 0.106 [0.026, 0.185], *p* = 0.0095), animal (*β* = 0.099 [0.018, 0.179], *p* = 0.0163), and vegetable (*β* = 0.128 [0.051, 0.206], *p* = 0.0013), naming tests (Tables 2, 3, S2, S3, S4). Additionally, BBB-crossing ARBs were associated with significantly better performance than BBB-crossing ACE-Is on the TMT-A, DSST, and vegetable naming tests (Tables 2, 3, S3).

## Discussion

Exposure to an ARB was associated with better performance on tests of memory and processing speed apparent over several years of follow-up when compared with exposure to an ACE-I in older adults with a diagnosis of AD dementia. In stratified analyses, this relative benefit was found to be significant only in non-carriers of the *APOE* ε4 allele. The relative benefits of ARBs to ACE-Is on memory performance were not dependent on intrinsic BBB-crossing properties of ARBs but were dependent on BBB penetrance for ACE-Is, such that the use of non-BBB-crossing ACE-Is was associated with significantly poorer outcomes. This study, incorporating epidemiological techniques to specifically compare two classes of RAAS-acting antihypertensive agents, demonstrates how these two heterogeneity factors underlie differences in the cognitive effects of ARBs and ACE-Is.

The main analyses revealed that ARB use was associated with better memory performance over time compared to ACE-I use, confirming results from several previous studies [25, 32, 36, 48] although other studies have reported the opposite or a null conclusion [49, 50]. Two recently published meta-analyses examined associations between antihypertensive drugs, cognitive decline, and dementia incidence, failing to identify significant differences between drug classes [12, 16]. Findings from stratified analyses suggest some possible reasons for these discrepancies, including factors related to the populations studied, and the drugs used.

Stratification by *APOE* ε4 carrier status revealed that the differences between ACE-Is and ARBs were driven by the subgroup of non-carriers of the ε4 allele. These results in people with AD dementia agree with a recent analysis by Tully et al., who examined associations between antihypertensive drug use and cognitive decline in non-demented older adults [35]. In that study, RAAS-targeting agents were associated with improved cognition relative to other antihypertensive drugs. Furthermore, those authors found that exposure to an ARB, but not to an ACE-I, was associated with better performance on a test of semantic verbal fluency and speed among *APOE* ε4 non-carriers. They found the opposite association among *APOE* ε4 carriers, whereby exposure to an ACE-I, but not to an ARB, was associated with better performance. Those findings are consistent with the present findings in AD patients that ARBs were associated with significantly better cognitive performance over time specifically in *APOE* ε4 non-carriers.

The present study identified associations between ARB use and performance on tests of memory, attention, verbal fluency and psychomotor processing speed in people with AD dementia. In contrast, Tully et al. found no significant differences between ARBs and ACE-Is in memory or attention in older adults without dementia. Therefore, it is possible that these differences are specific to people with clinical symptoms of AD pathology. The magnitude of anti-inflammatory and anti-oxidative benefits associated with ARBs through inverse agonism at the AT1R [66, 67] and upregulation of AT2R and AT4R activity [68, 69] might be amplified in AD. Furthermore, studies in vitro and in vivo have demonstrated that ARBs can increase degradation and clearance of Aβ peptides [70–72], though others in mouse models have observed no differences [73]. Moreover, a neuropathology study found that ARBs were associated with reduced Aβ plaque load, and a cross-sectional PET study yielded similar results with PiB binding [32, 33]. It has been suggested that preservation of angiotensin-converting enzyme-1 (ACE-1) function by ARBs, but not ACE-Is, may explain this difference, as ACE-1 has demonstrated a role in the prevention of Aβ aggregation and fibril formation in vitro [74, 75], although results from animal models have been highly variable [76–80].

The mechanistic reasons for the findings among ε4 non-carriers, but not among carriers, have yet to be explored. A recent study by Burnham et al. showed that *APOE* ε4 carriers and non-carriers did not differ in their rates of Aβ accumulation once a certain threshold had been reached, but that *APOE* ε4 carriers arrived at that threshold, on average, 15 years earlier than non-carriers [81]. In the current analysis, the mean age of APOE ε4 non-carriers was significantly greater than APOE ε4 carriers (78.4 years vs. 75.8 years). Thus, the ε4 carriers in our analysis were likely at a more advanced stage of amyloid, tau, or AD progression in general, at which point the preservation of ACE-1 function, upregulation of ACE-2 function, or other purported anti-inflammatory, anti-oxidative, and anti-amyloidogenic mechanisms related to ARB use may lack benefit. Previous evidence has associated treatment of hypertension, regardless of antihypertensive agent, with an exponential decrease in incident AD dementia risk and cognitive decline particularly among hypertensive APOE ε4 carriers, but not non-carriers [82, 83] which might suggest that the magnitude of cognitive benefit among ε4 carriers afforded by general blood pressure-lowering effects is larger and that the importance of other mechanistic differences between ARBs and ACE-Is may be reduced relative to those observed in ε4 non-carriers. Notably, however, systolic BP was included as a time-varying covariate in all models, so the effects reported are independent of blood pressure-lowering effectiveness, which itself (i.e., systolic blood pressure over time) was not found to be a significant predictor of cognitive decline. Genetics may also play a role in explaining the heterogeneity of observed effects, as one study found that *APOE* ε4 carriers with specific ACE genotypes benefitted more from ACE-Is in reducing cognitive decline than did non-carriers [84]; another study suggested that *APOE* and ACE genotypes may interact to confer risk of AD [85]. As *APOE* ε4 carriers are likely to have more severe AD pathology, our results might suggest that the benefits of ARBs in *APOE* ε4 non-carriers could relate to effects on the cerebrovasculature that are less important in carriers. Recently, ARB use was associated with a reduced rate of amyloid accumulation in the cortex relative to ACE-I use, and this effect was also smaller in APOE ε4 carriers [86], which would be consistent, for example, with the possibility of a more profound and relevant deficit in vascular amyloid clearance with vascular brain aging as a contributing cause of amyloidosis among APOE ε4 non-carriers who were older in this sample [87]. Further work to understand disease heterogeneity is warranted. Future studies should explore ACE polymorphisms as additional heterogeneity factors, particularly to determine if ACE polymorphisms might interact with drug exposures among *APOE* ε4 carriers [85, 88].

Generally, BBB penetration was associated with preserved cognitive performance; however, this effect was more prominent for ACE-Is than ARBs. In most cognitive domains assessed, use of a non-crossing ACE-I was associated with the poorest performance. Previous findings around BBB penetrance have been equivocal. Consistent with our findings, Ho et al. reported better memory performance in users of BBB-crossing RAAS medications, and specifically among users of BBB-crossing ARBs relative to users of non-BBB-crossing RAAS medications [30]. Tully et al. identified no significant associations between cognition and BBB penetration for either ACE-Is or ARBs, although marginally better scores were observed among users of centrally acting ARBs, consistent with the present study [35]. For ACE-Is, previous work has associated the use of BBB-penetrating ACE-Is with reduced cognitive decline and dementia incidence relative to the use of non-BBB-crossing ACE-Is and to other antihypertensive drugs, both in an AD [50, 89] and in a non-demented population [49]. Our observations provide a context for those findings. The importance of BBB penetration in determining ACE-I effects on cognition is further supported by previous work in animal models which showed that administration of perindopril, a BBB-crossing ACE-I, increased acetylcholine levels in the brain, while non-BBB-crossing ACE-Is did not [90]. The loss of cholinergic neurons in the basal forebrain is a defining characteristic of AD, and thus, this mechanism could underlie some of the cognitive benefits observed.

Our observation that there was no significant difference between BBB-crossing and non-crossing ARBs on memory and other cognitive domains might be explained by several factors. In a large epidemiological analysis of 819,941 older adults by Li et al. [48], the BBB-crossing ARBs candesartan and telmisartan were associated with significant dose-dependent reductions in dementia incidence; however, valsartan was not. An important limitation of our work is that a binary classification of ARBs and ACE-Is as BBB-crossing was made although these properties likely exist on a spectrum. Additionally, we acknowledge that there were some inconsistencies between the studies used to inform our classifications, and furthermore that data from animal models might not directly translate to the Alzheimer’s disease population considered in the present analysis. For example, one study found that losartan crosses the BBB [91], while another did not [92]. Although an absolute classification can be difficult to make, relative lipophilicity can be determined with greater certainty, for example, telmisartan was found at 10-fold higher concentrations than losartan in a rodent study [93]. Although telmisartan, candesartan, and valsartan are all considered to be BBB-crossing, many studies have suggested that telmisartan and candesartan specifically may offer disproportionate neuroprotective benefits via several potential mechanisms. Sequential studies of ARBs administered peripherally in rats to counter centrally administered angiotensin II identified these two agents as being the most brain-penetrating of all ARBs [39–41], and evidence from humans and non-human primates reinforces the BBB-crossing properties of telmisartan [94–96]. Valsartan is more commonly prescribed and constituted the majority of the BBB-crossing ARBs in our analyses; therefore, sample size precluded subgroup analyses of more highly lipophilic ARBs (i.e., telmisartan and candesartan). The present findings generally support the idea that BBB penetration, coupled with other drug-specific properties, might predict neuroprotective properties of ARBs, in addition to ACE-Is. Notably, there were no differences between crossing and non-crossing agents among ε4 carriers, although trends in the same direction remained. While there are multiple possible reasons for this, *APOE* ε4 has been associated with breakdown and increased permeability of the BBB [97], so it is possible that any additional benefits conferred by ARBs or ACE-Is that depend on their brain-penetrating properties are nullified in this group.

### Limitations

Strengths of the present work include a relatively large sample size, adjustment for confounding by indication through inverse probability of treatment weighting, and the selection of a homogeneous group of participants with AD dementia. We also acknowledge several limitations. Diagnoses were based primarily upon clinical criteria rather than biomarker and imaging data. Analyses could not be adjusted for drug dosage and drug adherence, because this information was not available in the NACC database. This might be important given evidence from Li et al. which suggested a dose-dependent effect of ARBs whereby higher doses were associated with lower dementia incidence [98]. Duration of drug exposure prior to entry into the NACC cohort was not available. Similarly, duration of hypertension and other vascular comorbidities (e.g. diabetes, hypercholesterolemia) were not available. Partially due to phasing out of the WMS-R LM IIA between UDS 2.0 and UDS 3.0, there was a high rate of loss to follow-up, such that fewer than half of all participants included in the analyses had more than one observation for the outcome of delayed recall; however, loss to follow-up was similar between exposure groups. It should be acknowledged that sampling bias might limit generalizability as participants enrolling into ADC cohorts typically have a higher level of education and socioeconomic status than the general population. Although we implemented inverse probability of treatment weighting to address potential confounding by indication, chronic kidney disease is a factor that may influence the prescription of ACE-Is vs. ARBs, but data on this diagnosis were not available. The findings do not provide mechanistic insight; further studies might examine biomarker data, including Aβ and tau, in addition to expression of RAAS components, such as ACE-1, ACE-2, and Mas, AT2R, AT4R, and other receptors in the regulatory arm of the pathway.

## Conclusion

This longitudinal analysis of participants with a diagnosis of AD dementia revealed that ARB exposure was associated with favorable cognitive outcomes relative to ACE-I exposure. This relationship was found to be heavily dependent on *APOE* genotype with ARBs yielding greatest benefits in *APOE* ε4 allele non-carriers. BBB penetration was identified as a significant moderator of the effects of ACE-Is, but not ARBs. The results consolidate previous findings. Further prospective studies should investigate the mechanisms by which ARBs may exert neuroprotective properties that may be beneficial against AD, in addition to how patient-level (e.g. genomic) factors might contribute to heterogeneity in drug response. Intervention trials to determine if ARBs may be preferable in patients with clinical symptoms of cognitive decline might also consider this opportunity for pharmacogenetics to optimize trial design and ultimately, prescription patterns.

## Supplementary Information

**Additional file 1.** Participant inclusion/exclusion flowchart, APOE ε4 carrier vs. non-carrier plots, and tables for secondary cognitive outcomes.

## Data Availability

The data analyzed in the current study were obtained from the National Alzheimer’s Coordinating Center (NACC). For further information on access to the database, please contact NACC (contact details can be found at https://www.alz.washington.edu/).

## Abbreviations

Aβ: Amyloid beta
AD: Alzheimer’s disease
ARB: Angiotensin II type-1 receptor blocker
AT_x_R: Angiotensin II type “X” Receptor
ACE-I: Angiotensin-converting enzyme inhibitor
*APOE*: Apolipoprotein E
BBB: Blood-brain barrier
NACC: National Alzheimer’s Coordinating Centre
RR: Rate ratio
RAAS: Renin-angiotensin-aldosterone system
TMT-A: Trail Making Test A
TMT-B: Trail Making Test B
