# Supplementary material for: The use of angiotensin-converting enzyme inhibitors vs. angiotensin receptor blockers and cognitive decline in Alzheimer’s disease: the importance of blood-brain barrier penetration and APOE ε4 carrier status
Source: Alzheimers Res Ther. 2021 Feb 11;13:43. doi: 10.1186/s13195-021-00778-8 (PMC7876820; doi:10.1186/s13195-021-00778-8)
Supplement: Supplementary file 1 — Additional file 1. Participant inclusion/exclusion flowchart, APOE ε4 carrier vs. non-carrier plots, and tables for secondary cognitive outcomes. [file 13195_2021_778_MOESM1_ESM.docx]

**Supplemental Materials**

**
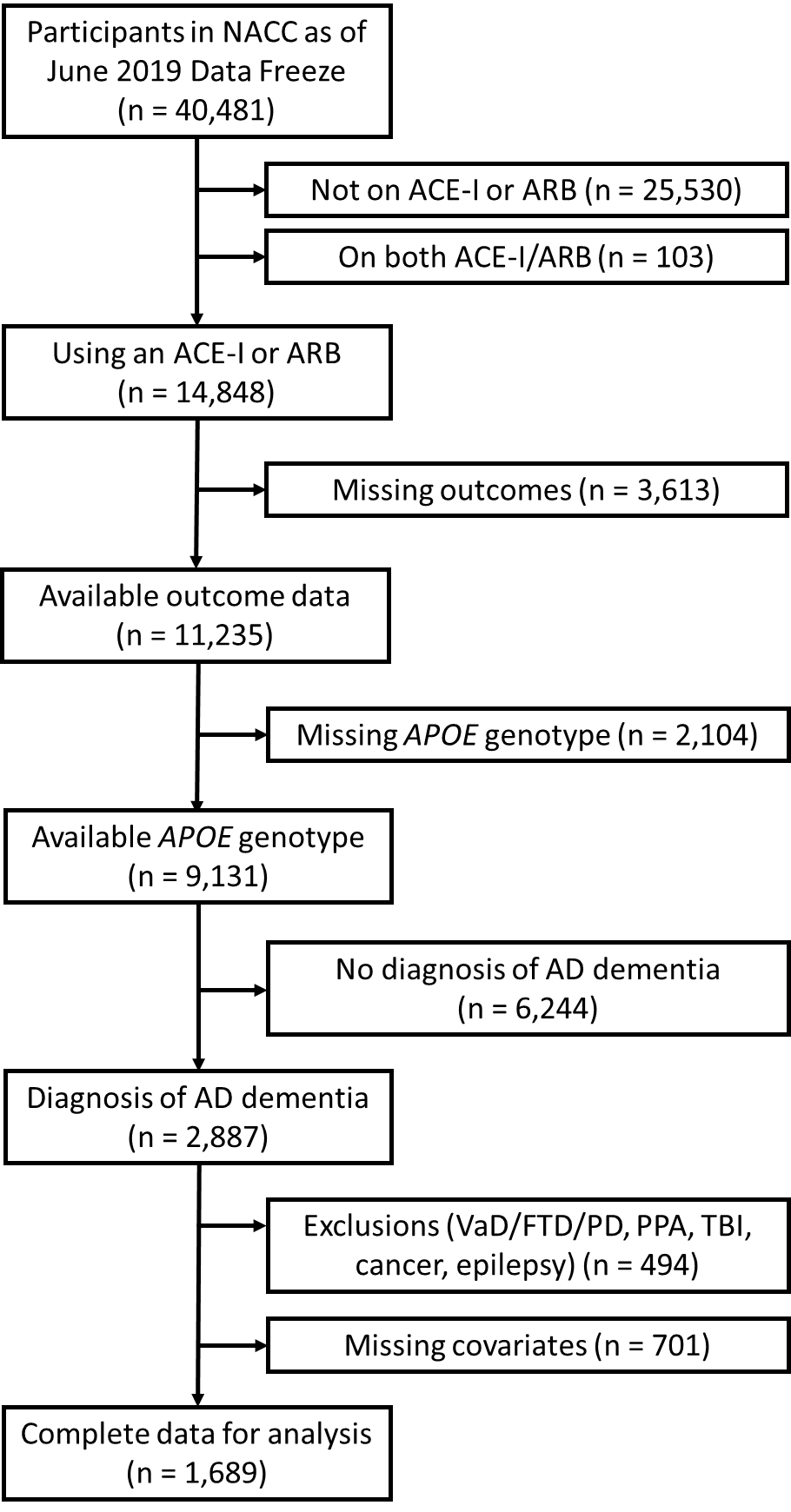
**

**Figure S1. Flowchart depicting process of participant selection from the NACC database for inclusion in analyses.**

**
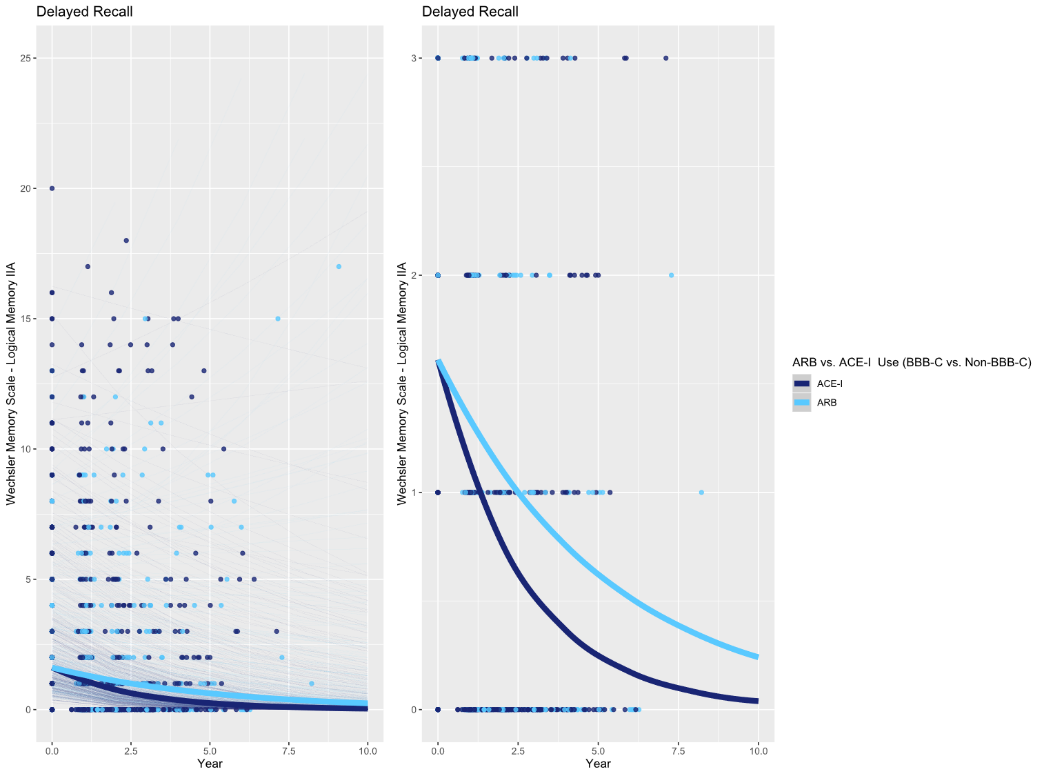
**

**
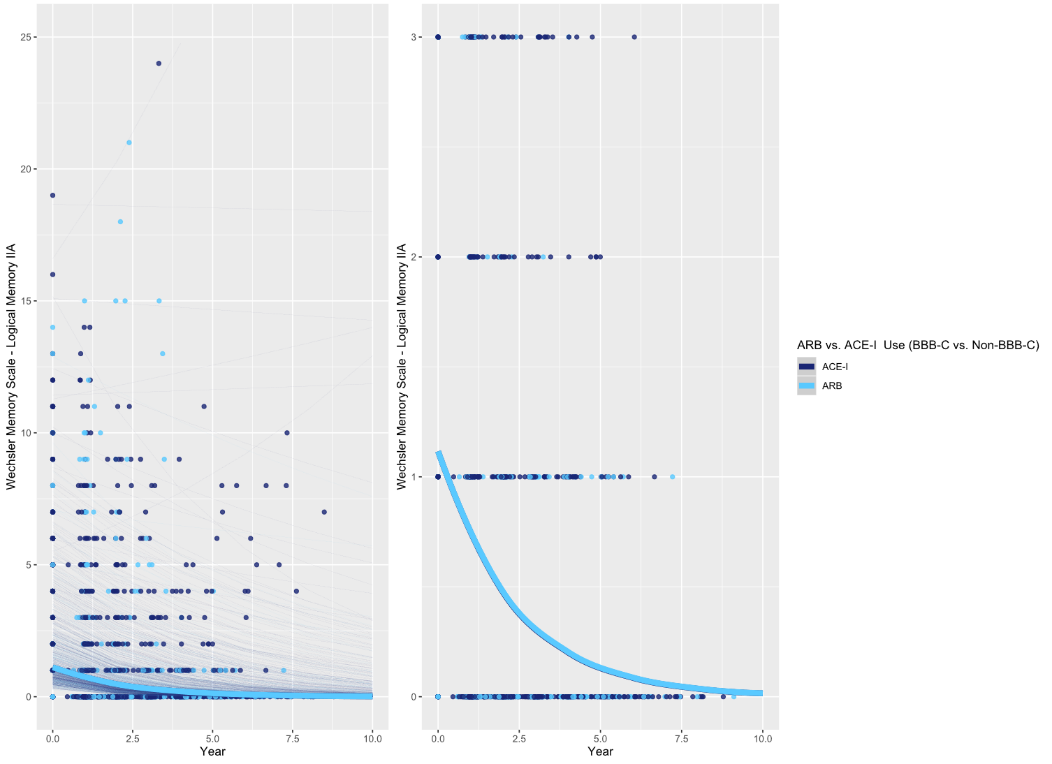
**

**Figure S2. Associations between ARB vs. ACE-I use and delayed recall performance over time in subjects with Alzheimer’s disease by APOE ε4 carrier status.**

**Top Left:** plot showing full range of outcome scores in APOE ε4 non-carriers; **Top** **Right:** plot with reduced y-axis cut-off, to better show differences between ARB and ACE-I groups in APOE ε4 non**-**carriers. **Bottom Left:** plot showing full range of outcome scores in APOE ε4 carriers; **Bottom** **Right:** plot with reduced y-axis cut-off, to better show differences between ARB and ACE-I groups in APOE ε4 carriers. Thick lines represent the total estimated association adjusted for covariates; thin lines represent estimated associations adjusted for covariates for each subject.

**Table S1. Relationships between Trail Making Test B performance and ACE-I vs. ARB use over time (n=1,315).**

|  | **Overall** | | | **APOE ε4 Non-Carriers** | | | **APOE ε4 Non-Carriers** | | |
| --- | --- | --- | --- | --- | --- | --- | --- | --- | --- |
|  | **β [95% CI]** | **z** | **p-value** | **β [95% CI]** | **z** | **p-value** | **β [95% CI]** | **z** | **p-value** |
| **TMT-B** |  |  |  |  |  |  |  |  |  |
| **ARBs vs.**  **ACE-Is** | 1.113  [-3.399, 5.625] | 0.48 | 0.6287 | 3.506  [-2.969, 9.982] | 1.06 | 0.2885 | 1.745  [-3.240, 6.731] | 0.69 | 0.4927 |
|  |  |  |  |  |  |  |  |  |  |
| **C-ARBs vs.**  **NC-ACE-Is** | -3.480  [-12.064, 5.103] | -0.80 | 0.4268 | -1.969  [-13.477, 9.539] | -0.34 | 0.7373 | 0.7394  [-9.430, 10.909] | 0.14 | 0.8867 |
|  |  |  |  |  |  |  |  |  |  |
| **C-ACE-Is vs. NC-ACE-Is** | -1.251  [-8.620, 6.117] | -0.33 | 0.7393 | -4.637  [-15.00, 5.722] | -0.88 | 0.3803 | 1.912  [-6.574, 10.397] | 0.44 | 0.6588 |
|  |  |  |  |  |  |  |  |  |  |
| **NC-ARBs vs.**  **NC-ACE-Is** | 2.326  [-5.622, 10.276] | 0.57 | 0.5662 | 1.594  [-9.802, 12.990] | 0.27 | 0.7839 | 4.553  [-4.484, 13.591] | 0.99 | 0.3234 |
|  |  |  |  |  |  |  |  |  |  |
| **C-ARBs vs. NC-ARBs** | -5.806  [-12.845, 1.232] | -1.62 | 0.1059 | -3.563  [-13.562, 6.435] | -0.70 | 0.4849 | -3.814  [11.547, 3.918] | -0.97 | 0.3336 |
|  |  |  |  |  |  |  |  |  |  |
| **C-ARBs vs.**  **C-ACE-Is** | -2.229  [-8.710, 4.260] | -0.67 | 0.5005 | 2.668  [-5.727, 11.064] | 0.62 | 0.5333 | -1.172  [-8.578, 6.234] | -0.31 | 0.7564 |
|  |  |  |  |  |  |  |  |  |  |

3-way APOE x ARB vs. ACE-I x Time interaction:

B = 1.762 seconds [-6.362, 9.885], z = 0.425, p = 0.6708

**Table S2. Relationships between Animal Naming Test performance and ACE-I vs. ARB use over time (n=1,686).**

|  | **Overall** | | | | **APOE ε4 Non-Carriers** | | | | **APOE ε4 Carriers** | | | |
| --- | --- | --- | --- | --- | --- | --- | --- | --- | --- | --- | --- | --- |
|  | **β [95% CI]** | t | **df** | **p-value** | **β [95% CI]** | t | **df** | **p-value** | **β [95% CI]** | t | **df** | **p-value** |
|  |  |  |  |  |  |  |  |  |  |  |  |  |
| **Animals** |  |  |  |  |  |  |  |  |  |  |  |  |
|  |  |  |  |  |  |  |  |  |  |  |  |  |
| **ARBs vs.**  **ACE-Is** | 0.011  [-0.040, 0.062] | 0.44 | 525.5 | 0.6627 | 0.051  [-0.023, 0.126] | 1.34 | 537.2 | 0.1794 | -0.003  [-0.062, 0.056] | -0.09 | 486.2 | 0.9286 |
|  |  |  |  |  |  |  |  |  |  |  |  |  |
| **C-ARBs vs.**  **NC-ACE-Is** | 0.036  [-0.021, 0.092] | 1.24 | 591.2 | 0.2169 | 0.099  [0.018, 0.179] | 2.41 | 640.5 | **0.0163** | 0.017  [-0.051, 0.085] | 0.05 | 523.8 | 0.6209 |
|  |  |  |  |  |  |  |  |  |  |  |  |  |
| **C-ACE-Is vs. NC-ACE-Is** | 0.068  [-0.006, 0.142] | 1.80 | 549.8 | 0.0725 | 0.082  [-0.027, 0.191] | 1.47 | 712.5 | 0.1428 | 0.064  [-0.024, 0.151] | 1.43 | 434.6 | 0.1545 |
|  |  |  |  |  |  |  |  |  |  |  |  |  |
| **NC-ARBs vs.**  **NC-ACE-Is** | 0.048  [-0.012, 0.109] | 1.56 | 547.3 | 0.1194 | 0.053  [-0.035, 0.141] | 1.18 | 694.7 | 0.2370 | 0.042  [-0.030, 0.113] | 1.14 | 443.5 | 0.2529 |
|  |  |  |  |  |  |  |  |  |  |  |  |  |
| **C-ARBs vs.**  **NC-ARBs** | -0.007  [-0.059, 0.045] | -0.27 | 728.3 | 0.7910 | 0.051  [-0.019, 0.123] | 1.42 | 815.3 | 0.1560 | -0.020  [-0.080, 0.040] | -0.66 | 680.5 | 0.5112 |
|  |  |  |  |  |  |  |  |  |  |  |  |  |
| **C-ARBs vs.**  **C-ACE-Is** | -0.007  [-0.054, 0.040] | -0.29 | 622.1 | 0.7681 | 0.047  [-0.020, 0.114] | 1.38 | 563.6 | 0.1673 | -0.023  [-0.078, 0.032] | -0.82 | 614.5 | 0.4130 |
|  |  |  |  |  |  |  |  |  |  |  |  |  |

3-way APOE x ARB vs. ACE-I x Time interaction:

β = 0.035 [-0.027, 0.097], t = 1.11, p = 0.2694

**Table S3. Relationships between Vegetable Naming Test performance and ACE-I vs. ARB use over time (n=1,680).**

|  | **Overall** | | | | **APOE ε4 Non-Carriers** | | | | **APOE ε4 Carriers** | | | |
| --- | --- | --- | --- | --- | --- | --- | --- | --- | --- | --- | --- | --- |
|  | **β [95% CI]** | t | **df** | **p-value** | **β [95% CI]** | t | **df** | **p-value** | **β [95% CI]** | t | **df** | **p-value** |
|  |  |  |  |  |  |  |  |  |  |  |  |  |
| **Vegetables** |  |  |  |  |  |  |  |  |  |  |  |  |
|  |  |  |  |  |  |  |  |  |  |  |  |  |
| **ARBs vs.**  **ACE-Is** | -0.002  [-0.051, 0.046] | -0.10 | 417.1 | 0.9232 | 0.075  [0.003, 0.147] | 2.04 | 482.3 | **0.0420** | -0.027  [-0.084, 0.029] | -0.95 | 400.4 | 0.3451 |
|  |  |  |  |  |  |  |  |  |  |  |  |  |
| **C-ARBs vs.**  **NC-ACE-Is** | 0.027  [-0.026, 0.081] | 1.00 | 490.1 | 0.3172 | 0.128  [0.051, 0.206] | 3.24 | 566.0 | **0.0013** | -0.011  [-0.075, 0.054] | -0.32 | 442.9 | 0.7470 |
|  |  |  |  |  |  |  |  |  |  |  |  |  |
| **C-ACE-Is vs. NC-ACE-Is** | 0.063  [-0.007, 0.134] | 1.77 | 451.0 | 0.0770 | 0.092  [-0.013, 0.197] | 1.72 | 663.6 | 0.0866 | 0.042  [-0.041, 0.124] | 0.98 | 351.3 | 0.3260 |
|  |  |  |  |  |  |  |  |  |  |  |  |  |
| **NC-ARBs vs.**  **NC-ACE-Is** | 0.033  [-0.025, 0.090] | 1.12 | 433.3 | 0.2624 | 0.069  [-0.015, 0.154] | 1.61 | 621.9 | 0.1088 | 0.011  [-0.056, 0.079] | 0.33 | 350.7 | 0.7400 |
|  |  |  |  |  |  |  |  |  |  |  |  |  |
| **C-ARBs vs. NC-ARBs** | -0.002  [-0.051, 0.048] | -0.07 | 565.2 | 0.9451 | 0.066  [-0.002, 0.135] | 1.90 | 706.1 | 0.0577 | -0.021  [-0.077, 0.036] | -0.72 | 529.1 | 0.4711 |
|  |  |  |  |  |  |  |  |  |  |  |  |  |
| **C-ARBs vs.**  **C-ACE-Is** | -0.013  [-0.058, 0.033] | -0.55 | 514.9 | 0.5833 | 0.070  [0.005, 0.134] | 2.12 | 490.9 | **0.0344** | -0.037  [-0.090, 0.016] | -1.38 | 517.3 | 0.1697 |
|  |  |  |  |  |  |  |  |  |  |  |  |  |

3-way APOE x ARB vs. ACE-I x Time interaction:

β = 0.066 [0.007, 0.126], t = 2.18, **p = 0.0298**

**Table S4. Relationships between Boston Naming Test performance and ACE-I vs. ARB use over time (n=1,671).**

|  | **Overall** | | | | **APOE ε4 Non-Carriers** | | | | **APOE ε4 Carriers** | | | |
| --- | --- | --- | --- | --- | --- | --- | --- | --- | --- | --- | --- | --- |
|  | **β [95% CI]** | t | **df** | **p-value** | **β [95% CI]** | t | **df** | **p-value** | **β [95% CI]** | t | **df** | **p-value** |
|  |  |  |  |  |  |  |  |  |  |  |  |  |
| **Boston** |  |  |  |  |  |  |  |  |  |  |  |  |
|  |  |  |  |  |  |  |  |  |  |  |  |  |
| **ARBs vs.**  **ACE-Is** | 0.011  [-0.030, 0.052] | 0.54 | 604.9 | 0.5900 | 0.045  [-0.021, 0.110] | 1.33 | 600.2 | 0.1838 | -0.003  [-0.053, 0.047] | -0.11 | 667.6 | 0.9104 |
|  |  |  |  |  |  |  |  |  |  |  |  |  |
| **C-ARBs vs.**  **NC-ACE-Is** | 0.017  [-0.029, 0.062] | 0.72 | 626.4 | 0.4724 | 0.038  [-0.030, 0.105] | 1.10 | 732.9 | 0.2723 | 0.010  [-0.047, 0.068] | 0.35 | 597.68 | 0.7272 |
|  |  |  |  |  |  |  |  |  |  |  |  |  |
| **C-ACE-Is vs. NC-ACE-Is** | 0.024  [-0.036, 0.085] | 0.79 | 570.3 | 0.4300 | -0.014  [-0.103, 0.076] | -0.30 | 751.3 | 0.7672 | 0.047  [-0.030, 0.124] | 1.19 | 492.2 | 0.2332 |
|  |  |  |  |  |  |  |  |  |  |  |  |  |
| **NC-ARBs vs.**  **NC-ACE-Is** | 0.025  [-0.025, 0.074] | 0.98 | 610.3 | 0.3274 | 0.014  [-0.059, 0.087] | 0.39 | 758.5 | 0.6989 | 0.033  [-0.029, 0.095] | 1.04 | 539.6 | 0.3006 |
|  |  |  |  |  |  |  |  |  |  |  |  |  |
| **C-ARBs vs. NC-ARBs** | -0.005  [-0.045, 0.035] | -0.26 | 1039.4 | 0.7978 | 0.025  [-0.031, 0.081] | 0.88 | 1296.1 | 0.3816 | -0.019  [-0.065, 0.027] | -0.80 | 1073.6 | 0.4242 |
|  |  |  |  |  |  |  |  |  |  |  |  |  |
| **C-ARBs vs.**  **C-ACE-Is** | 0.001  [-0.036, 0.039] | 0.07 | 737.8 | 0.9447 | 0.046  [-0.011, 0.103] | 1.58 | 729.7 | 0.1135 | -0.019  [-0.064, 0.025] | -0.85 | 838.4 | 0.3978 |
|  |  |  |  |  |  |  |  |  |  |  |  |  |

3-way APOE x ARB vs. ACE-I x Time interaction:

β = 0.031 [-0.023, 0.084], t = 1.12, p = 0.2613
